# Knowledge and attitude toward eye disorders in children among pediatricians and family physicians: a survey study

**DOI:** 10.1186/s12886-023-02832-5

**Published:** 2023-03-07

**Authors:** Reem M. Hersi, Nada K. Naaman, Amer M. Alghamdi, Wejdan A. Alnahdi, Ziad M. Bukhari, Hashem S. Almarzouki

**Affiliations:** 1 College of Medicine, King Saud bin Abdulaziz University for Health Sciences, Jeddah, Saudi Arabia; 2 Department of Ophthalmology, College of Medicine, King Saud University, Riyadh, Saudi Arabia; 3 Department of Ophthalmology, King Abdulaziz Medical City, Ministry of National Guard Health Affairs, Jeddah, Saudi Arabia; 4 King Abdullah International Medical Research Center, Jeddah, Saudi Arabia

**Keywords:** Knowledge, Attitude, Eye disorders, Children, Pediatrics, Pediatrician, Family physician, Congenital glaucoma, Leukocoria, Red eye

## Abstract

**Background:**

Vision-related disorders are common in children. Therefore, eye examination and thorough visual assessment by first-contact physicians are crucial in children. This study aimed to evaluate the knowledge of and attitude toward children’s eye disorders among pediatricians and family physicians in the Ministry of National Guard Health Affairs-Western Region (MNGHA-WR) of Saudi Arabia.

**Methods:**

In this observational, cross-sectional study, we used a self-administered, web-based questionnaire. The sample size was calculated to be 148 pediatricians and family physicians (of 240 in total) currently working at MNGHA-WR. The first section of the questionnaire dwelled on demographics, while the second section addressed the physician’s knowledge of and attitude toward commonly encountered ophthalmological pathologies in children. Data collected were entered into Microsoft Excel and then transferred to IBM SPSS version 22 for statistical analysis.

**Results:**

A total of 148 responses (92 family physicians and 56 pediatricians) were received. Most of the participants were residents or staff physicians (n = 105, 70.9%). The mean knowledge score of the respondents was 54.67% ± 14.5%. Participants’ knowledge was further subclassified using Bloom’s original cutoff points into high (n = 4, 2.7%), moderate (n = 53, 35.8%), and low (n = 91, 61.5%) levels of knowledge. Regarding practices, 120 (81%) participants performed ophthalmic examinations; however, only 39 (26.4%) conducted routine examinations as part of every child’s visit. Fundus examinations were performed by 25 (16.9%) physicians. A significant deficiency in knowledge was noted in those with < 1 year of work experience (P = 0.014). Although statistically not significant (P = 0.052), family physicians possessed better knowledge than pediatricians regarding children’s eye disorders. On the contrary, more pediatricians performed eye examinations than family physicians (P = 0.015). The male sex was also associated with higher rates of eye examination (P = 0.033).

**Conclusion:**

An unsatisfactory level of knowledge of eye disease among participating doctors was reported. The proportion was significantly higher among residents and staff physicians. Therefore, awareness efforts should be incorporated in both family medicine and pediatrics residency programs to limit the number of cases of ocular disorders going undiagnosed in children.

**Supplementary Information:**

The online version contains supplementary material available at 10.1186/s12886-023-02832-5.

## Background

Vision-related disorders are common among children. It is estimated that 19 million children below the age of 14 years worldwide are visually impaired. A major cause of such impairment is uncorrected errors of refraction (43%) [1]. In Jazan, Saudi Arabia, 36.9% and 26.5% of children suffer from strabismus and refractive errors, respectively [2]. Another study conducted in Dammam, Saudi Arabia, found that amblyopia is present in 9.1% of children [3].

Various ocular diseases such as retinoblastoma, congenital cataract, congenital glaucoma, and some other retinal abnormalities could lead to marked visual impairment in children, which is considered a significant public health concern because it negatively affects the children in question, their families, and society. Fortunately, these ocular conditions could be detected during a child’s visit to a pediatrician or a family physician or via early referral to a specialized ophthalmologist [4]. Therefore, early detection through eye examinations and thorough visual assessment with immediate management are crucial and might save children from life-long visual impairments.

Moreover, to avoid any permanent visual disability, eye examinations should be performed in children from the neonatal period and at every follow-up visit during infancy [4]. Further, visual acuity should be assessed as early as three years of age [5, 6]. First-contact physicians should also pay special attention to patients at higher risk of ocular diseases (those with a positive family history, consanguinity, prematurity, or neurological deficits). Therefore, they should be familiar with the available screening guidelines to detect ocular structural abnormalities, refractive errors, strabismus, and congenital cataract. Accordingly, they should be aware of joint eye examination guidelines [5, 7]. A few studies have assessed the knowledge and practice of non-ophthalmology physicians who manage specific children’s eye diseases in different regions of Saudi Arabia and other countries [8–12]; however, our study reports the knowledge and practice of multiple eye diseases in children among pediatricians and family physicians in the western region of Saudi Arabia.

## Methods

In this study, we used a descriptive observational cross-sectional design, and participants were invited to fill out a web-based questionnaire. Using a nonprobability convenient sampling technique, the study included all pediatric and family medicine consultants, specialists, and residents working at MNGHA-WR. Family medicine and pediatrics physicians who were working at institutions other than MNGHA-WR were excluded from the study. All 116 pediatricians and 124 family physicians (a total of 240) were invited to participate in this study. Using a confidence level of 95% and a 5% margin of error, the target sample size was calculated to be 148 physicians using the “Raosoft” software program (Raosoft, Inc.).

The questionnaire by Wanyama et al. (2013) and another one by Ababneh et al. (2021) addressing knowledge and attitude toward eye disorders among pediatricians were combined to meet this study’s objectives [9, 13]. Participants were invited to complete a structured, self-administered survey questionnaire sent through the hospital’s message center. Participants gave their informed consent to participate before completing the questionnaire. The questionnaire’s first section comprised demographic data, such as age, sex, duration of practice, and position at work/seniority. The second part addressed the physician’s knowledge of commonly encountered ophthalmological pathologies and his/her approach toward them. An overview of the consent form and questionnaire is presented in Appendix 1.

Data collected from the questionnaire were entered into Microsoft Excel and then exported to IBM SPSS Statistics version 22 (IBM Corp., Armonk, NY, USA) for statistical analysis. Quantitative data were presented using mean values and standard deviations, whereas qualitative data were presented using frequencies and percentages. We used the chi-square test to compare categorical variables. P-values of < 0.05 were considered statistically significant.

Scoring of the question is limited to the questions examining the knowledge of the participants. There are a total of 11 questions in the knowledge section; some of them will let the participants to select more than one response because there may be more than one correct answer. Each correct answer will give the participant one point, while incorrect responses receive zero points. The cumulative score of each participant will then be converted into a percentage. Finally, every participant’s score will be allocated based on original Bloom’s cutoff points into good knowledge (80–100%), moderate knowledge (60–80%), and poor knowledge (< 60%).

## Results

A total of 148 responses were received. The study participants were aged 24–61 years, with a mean of 31 ± 8 years. The sample included 79 (53.1%) female physicians and 69 (46.6%) male physicians. The respondents were 92 (62.2%) family physicians and 56 (37.8%) pediatricians. Most of the physicians were residents or staff physicians (n = 105, 70.9%), and only 43 (29.1%) participants were consultants, assistant consultants, fellows, or specialists. Detailed distributions of the study population by working status, duration of practice, and attendance of pediatric ophthalmic conferences are shown in Table 1.

**Table 1:** Distribution of participants by current working status, current year of residency, length of practicing, and pediatric ophthalmic conference attendance

Variable	n (%)
Current working status	
Pediatric consultants/assistant consultants	14 (9.5%)
Pediatric fellows/specialists	0 (0%)
Pediatric staff physicians	2 (1.4%)
Pediatric residents	40 (27%)
Family medicine consultants / assistant consultants	27 (18.2%)
Family medicine fellows / specialists	2 (1.4%)
Family medicine staff physicians Family medicine residents	1 (0.7%) 62 (41.9%)
**Current year of residency**	
First year R1	40 (27%)
Second year R2	25 (16.9%)
Third year R3	20 (13.5%)
Fourth year R4	17 (11.5%)
Total	102 (68.9%)
**Length of practicing**	
<1 year	43 (29.1%)
1–4 years	62 (41.9%)
5–10 years	12 (8.1%)
> 10 years	31 (20.9%)
**Attending a pediatric ophthalmic conference**	
Yes	27 (18.2%)
No	121 (81.8%)

The knowledge section of the questionnaire focused on specific pediatric eye disorders, including refractive errors, strabismus, leukocoria, congenital glaucoma, and retinopathy of prematurity (ROP). Regarding the causes of painful red eyes, corneal traumatic abrasion was the most commonly reported cause (n = 129, 87.2%), followed by uveitis (n = 103, 69.6%). Concerning questions about strabismus, 42 (28.4%) participants did not know if refractive errors could cause strabismus, whereas 91 (61.5%) reported that it is true that strabismus can be caused by refractive errors. Regarding the causes of leukocoria, retinoblastoma was reported by 129 (87.2%), and cataract was reported by 81 (54.7%). Only 5 (3.4%) agreed wrongly that leukocoria is a normal variation in children. Participants’ knowledge about ophthalmic eye disorders in pediatrics regarding risk factors, causes, and clinical presentations is shown in Table 2.

**Table 2:** Distribution of participants by knowledge of specific pediatric eye disorders

Variable	n (%)
*When should an ophthalmologist see a child?	
No need if there are no symptoms of an eye disorder	22 (14.9%)
All new born	32 (21.6%)
During regular well-baby visits	24 (16.2%)
**Should have vision screening at least once before kindergarten**	102 (68.9%)
I don’t know	3 (2%)
*Which of the following can cause red painful eye disease in children?	
**Conjunctivitis**	86 (58.1%)
**Allergy**	49 (33.1%)
**Uveitis**	103 (69.6%)
**Corneal abrasion / trauma**	129 (87.2%)
**Cataract**	4 (2.7%)
**Glaucoma**	36 (24.3%)
Squint	2 (1.4%)
*Which of the following can cause leukocoria?	
**Cataract**	81 (54.7%)
Glaucoma	21 (14.2%)
**Retinoblastoma**	129 (87.2%)
Toxocariasis	18 (12.2%)
**Advanced retinal disorder**	19 (12.8%)
**Leukocoria could be?**	
**Sight-threatening**	68 (45.9%)
**Life-threatening**	75 (50.7%)
Normal variation between children	5 (3.4%)
**Children of any age may have refractive errors and may need glasses.**	
**True**	126 (85.1%)
False	5 (3.4%)
I don’t know	17 (11.5%)
**Refractive errors can cause squint.**	
**True**	91 (61.5%)
False	15 (10.1%)
I don’t know	42 (28.4%)
*Which of the following give a clue that a child may have true squint?	
**Eye deviation**	114 (77%)
**Face turn**	31 (20.9%)
**Anomalous head posture**	41 (27.7%)
Epicanthal fold	6 (4.1%)
Wide nasal bridge	1 (0.7%)
*What are the concerns in a child with squint?	
**Cosmetically not acceptable**	30 (20.3%)
**Amblyopia**	123 (83.1%)
**Underlying central cause**	76 (51.4%)
**Squint can be treated by?**	
**Glasses**	92 (62.2%)
**Surgical repair**	37 (25%)
**Spontaneously resolving as a child grows**	19 (12.8%)
*Which of the following is a sign of CG?	
**Watering**	21 (14.2%)
Leukocoria	58 (39.2%)
**Large cornea**	65 (43.9%)
**Hazy cornea**	76 (51.4%)
**Red eye**	30 (20.3%)
**Which of the following may be a risk factor of ROP?**	
Birth weight < 1500 g	0 (0%)
Gestational age ≤ 32 weeks	0 (0%)
Premature baby with comorbidities	0 (0%)
**All of the above**	148 (100%)

*More than one choice

The practices of pediatricians and family medicine physicians, focusing on the examination and management of pediatric eye disorders, were assessed. Most participants (81%) performed ophthalmic examinations. Those who performed examinations were asked about the timing of the examination; 86 (58.1%) conduct an examination only when a caregiver complained of an eye problem, 68 (45.9%) routinely performed examinations at birth, 39 (26.4%) conducted examination routinely during every child visit, and 12 (8.1%) only conducted annual examinations. Of the types of tests performed, the red reflex was assessed by 112 (75.7%) participants, the pupillary reflex by 88 (59.5%) participants, visual acuity by 69 (46.6%) participants, and extraocular muscle function by 69 (46.6%) participants. Moreover, 25 (16.9%) physicians performed fundus examinations.

Those who did not perform eye examinations on children were asked why they did not do so. Eight (5.4%) of them reported that they do not usually have enough time, 11 (7.4%) said they do not often have the required equipment, and 14 (9.5%) said they do not know how to perform such an examination. Moreover, 9 (6.1%) stated that children are uncooperative, and 5 (3.4%) stated that it is not related to their specialty. Participants were also asked about the management of specific pediatric eye disorders (Table 3**)**.

**Table 3:** Distribution of participants based on management of specific pediatric eye disorders

Variable	n (%)
How do you manage a child with a painful red eye?	
Refer immediately to an ophthalmologist	76 (51.5%)
Give eye drops and refer immediately	19 (12.8%)
Give eye drops and refer after 3 days if no improvement	42 (28.4%)
Management depend on the cause	10 (6.8%)
I don’t know	1 (0.7%)
*If you chose “eye drops” for the management of painful red eye, please specify the type.	
Artificial tears	9 (6.1%)
Antibiotics	22 (14.9%)
Steroid	3 (2%)
Antihistamine	7 (4.7%)
**How do you manage a child with leukocoria?**	
Refer immediately to an ophthalmologist	144 (97.3%)
Give eye drops	1 (0.7%)
Follow-up; if no improvement, refer to an ophthalmologist	3 (2%)
**How do you manage a child with neonatal conjunctivitis?**	
Refer immediately to an ophthalmologist	68 (45.9%)
Give eye drops	75 (50.7%)
Follow-up; if no improvement, refer to an ophthalmologist	5 (3.4%)
Admit the patient immediately	1 (0.7%)
It is a self-limiting benign condition	1 (0.7%)
*If you chose “eye drops” for the management of neonatal conjunctivitis, please specify the type.	
Ofloxacin	14 (9.5%)
Gentamicin	7 (4.7%)
Erythromycin	9 (6.1%)
Chloramphenicol	1 (0.7%)
Unspecified antibiotic	9 (6.1%)
Artificial tears	4 (2.7%)
**How do you manage a child with a squint?**	
Refer immediately to an ophthalmologist	117 (79.1%)
Follow-up; if no improvement, refer to an ophthalmologist	26 (17.6%)
Brain imaging	5 (3.4%)
**How do you manage a child with CG?**	
Refer immediately to an ophthalmologist	145 (98%)
Give eye drops	0 (0%)
Follow-up; if no improvement, refer to an ophthalmologist	3 (2%)
**When should a child with congenital cataract be referred to an ophthalmologist?**	
When vision drops	3 (2%)
When caregiver request referral	2 (1.4%)
**Immediately**	143 (96.6%)
**When will you refer a premature baby for ROP screening?**	
After NICU discharge	22 (14.9%)
**4–6 weeks after birth or at 32 weeks, whichever later**	54 (36.5%)
At birth	34 (23%)
I don’t know	38 (25.7%)

*more than one choice

NICU, neonatal intensive care unit; ROP, retinopathy of prematurity

**Fig. 1:**
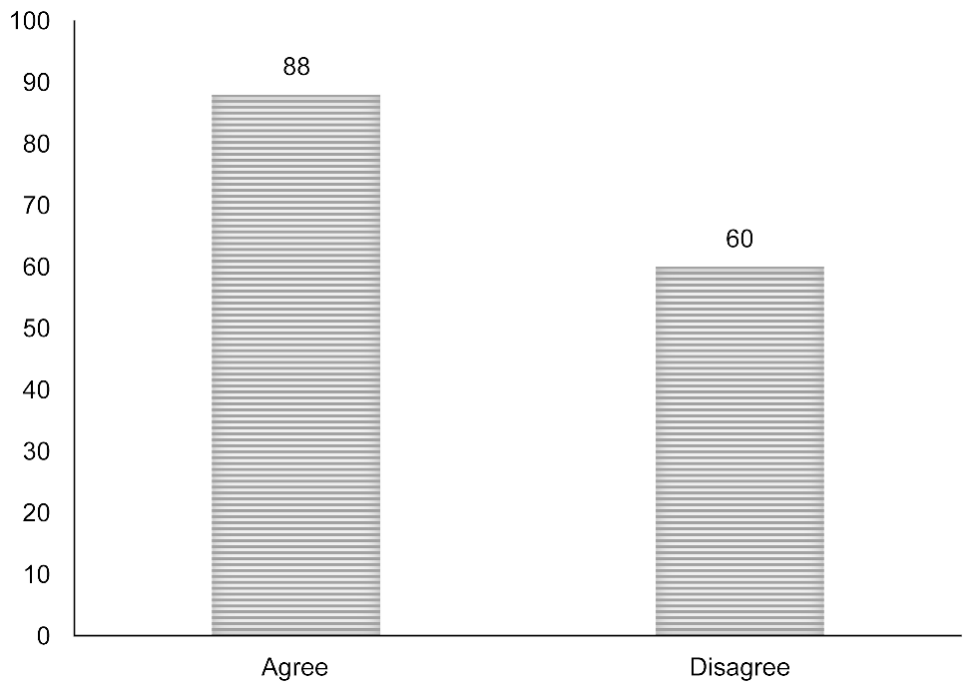
Your training adequately equips you to diagnose, manage, and refer children with eye diseases

The mean knowledge score of the respondents was 54.67% ± 14.5%. Participants’ knowledge was further classified using Bloom’s original cutoff points into high, moderate, and low levels of knowledge (Table 4**)**. Participants were asked if their training adequately equipped them to diagnose, manage, and refer children with eye diseases, and 60 (40.5%) of them disagreed (Fig. 1**)**.

**Table 4:** Knowledge level of responders according to Bloom’s cutoff points

Cut off point (n = 148)	n (%)
Low level of knowledge (> 60%)	91 (61.5%)
Moderate level of knowledge (60–80%)	53 (35.8%)
High level of knowledge (80–100%)	4 (2.7%)

Using Bloom’s original cutoff points, knowledge score categories were further categorized into satisfactory knowledge (high and moderate levels of knowledge) and unsatisfactory knowledge (low level of knowledge). This categorization was compared with the sociodemographic factors of the respondents (Table 5), and a significant knowledge deficiency was found in those with < 1 year of practice. We found that doctors with at least ten years of work experience had significantly more satisfactory knowledge than those with less than ten years of work experience (P = 0.014). In addition, consultants, associate consultants, specialists, and fellows had more satisfactory knowledge (46.5%) than residents and staff physicians (38.5%) (P = 0.201). Furthermore, the sociodemographic factors were compared with the practice of examination to look for any significant associations (Table 6**)**. Although statistically not significant (P = 0.052), family physicians possessed better knowledge than pediatricians on eye disorders. On the contrary, significantly more pediatricians performed eye examinations than family physicians (P = 0.015). Male physicians were also more likely to perform examinations (P = 0.033).

**Table 5:** Association between the level of knowledge among responders and sociodemographic factors

Variable	Overall n = 148	Satisfactory* knowledge n = 57	Unsatisfactory knowledge n = 91	P value**
**Sex** Male Female	69 79	25 (36.2%) 32 (40.5%)	44 (63.8%) 47 (59.5%)	0.594
**Length of practicing** <1 year 1–4 years 5–10 years >10 years	43 62 12 31	8 (18.6%) 30 (48.4%) 6 (50%) 13 (41.9%)	35 (81.4%) 32 (51.6%) 6 (50%) 18 (58.1%)	**0.014**
**Position** Consultants, associate consultants, specialists, or fellows Residents or staff physicians	43 105	20 (46.5%) 37 (38.5%)	23 (53.5%) 68 (64.8%)	0.201
**Specialty** Pediatric Family medicine	56 92	16 (28.6%) 41 (44.6%)	40 (71.4%) 51 (55.4%)	0.052

*Satisfactory: high and moderate level of knowledge

**Chi-square test

**Table 6:** Association between practicing eye examination and sociodemographic factors

Variable	Overall n = 148	Perform examination n = 120	Do not perform examination n = 28	P value
Sex Male Female	69 79	61 (88.4%) 59 (74.7%)	8 (11.6%) 20 (25.3%)	0.033
**Length of practicing** <1 year 1–4 years 5–10 years >10 years	43 62 12 31	34 (79.1%) 51 (82.3%) 11 (91.7%) 24 (77.4%)	9 (20.9%) 11 (17.7%) 1 (8.3%) 7 (22.6%)	0.725
**Position** Consultants, associate consultants, specialists, or fellows Residents or staff physicians	43 105	35 (81.4%) 85 (81%)	8 (18.6%) 20 (19%)	0.950
**Specialty** Pediatric Family medicine	56 92	51 (91.2%) 69 (75%)	5 (8.9%) 23 (25%)	**0.015**

## Discussion

Pediatricians and family physicians should be knowledgeable enough to recognize common pediatric eye disorders and early presentations because some eye diseases might be sight-threatening or life-threatening. In addition, children may lack the capability or the insight to voice out their ocular complaints, which may delay the proper healthcare and management. Thus, well-trained physicians should catch these disorders early enough. In terms of knowledge, participants’ medical understanding of eye diseases was inadequate, particularly among junior residents and staff doctors of both disciplines. The expertise and abilities of consultants, associate consultants, specialists, and fellows were satisfactory.

To begin with, participants had an excellent insight into recognizing the causes of painful red eyes in children. The most commonly reported causes were corneal traumatic abrasion (87.2%), uveitis (69.6%), and glaucoma (24.3%). Although conjunctivitis will probably cause a painless red eye, 85.1% of our participants stated inaccurately that it causes painful red eyes. These results are in line with the findings of other studies conducted in Jordan and Kenya [8, 14]. In comparison to a study conducted in the United States, in which 55% of the general practitioners were willing to prescribe eye drops or ointments to children with painful red eyes, more than half of our physicians (51.5%) were willing to refer the patient immediately to an ophthalmologist [13]. Some of the remaining participants (12.8%) chose to start their management by giving eye drops and then referring immediately or referring after three days if no improvement was observed (28.4%). The majority of eye drops prescribed by our physicians were antibiotics (14.9%), artificial tears (6.1%), antihistamines (4.7%), or steroids (2%) depending on the suspected cause. Additionally, regarding neonatal conjunctivitis, physicians’ approach was either referring the patient immediately to an ophthalmologist or giving eye drops such as ofloxacin (9.5%), erythromycin (6.1%), unspecified antibiotics (6.1%), gentamicin (4.7%), chloramphenicol (2.7%), or artificial tears (0.7%).

Knowledge of the causes of leukocoria was satisfactory, as 87.2% of the participants could recognize the most life-threatening and serious cause; i.e., retinoblastoma, and 54.7% identified the most common cause, i.e., cataracts. This was much higher than the finding of a study conducted in Brazil by Manica et al., wherein retinoblastoma was reported only by 37% of their participants [12]. Other reported causes were toxocariasis and advanced retinal disorders (12.2% and 12.8%, respectively). Although glaucoma is not a cause of leukocoria [15], 14.2% have reported incorrectly that it could be the etiology. Almost half of the physicians knew that leukocoria could be life- and/or sight-threatening, and a few (3.4%) of them believed (wrongly) that it could be a normal variation in children. Additionally, nearly all physicians (n = 144, 97.3%) would immediately refer a case of leukocoria/retinoblastoma or congenital cataract (n = 143, 96.6%) to an ophthalmologist for prompt management. On the contrary, a study conducted in the Qassim region of Saudi Arabia found that only 69% of nonophthalmic health professionals knew the correct action when encountering a retinoblastoma case; moreover, they had less than the desired knowledge about retinoblastoma [16].

Although nearly all of the participants of the study conducted in Kenya (98.4%) knew that true strabismus (i.e., strabismus) could be caused by refractive errors, only 61.5% of our participants reported that a refractive error could be a cause. Thus, the identification of those patients with refractive errors by healthcare professionals, parents, and teachers is important to prevent future consequences such as refractive strabismus or amblyopia. Signs of pseudostrabismus, including a wide nasal bridge and epicanthal fold, should be distinguished from those of true strabismus. In our study, participants showed great awareness of that distinction, with only 5% of them having difficulties with the concept. Because of their concern that the strabismus might lead to amblyopia (83.1%) or originate from a central cause (51.4%), most of the physicians were in favor of immediately referring a child with strabismus to an ophthalmologist (79.1%) or at least follow-up and refer when no improvement is seen (17.6%). A few of them reported that they might order brain imaging such as computed tomography or magnetic resonance imaging before referring to an ophthalmologist, which is an advanced step and should be done in collaboration between those physicians and ophthalmologists in certain types of pediatric strabismus [17]. Commonly, strabismus does not resolve spontaneously as the child grows, which was stated incorrectly by 12.8% of the participants. Instead, it is corrected either surgically or by wearing spectacles. Although most of the participating physicians were aware of the proper approach to a child with strabismus, which was reassuring, more effort should be directed toward being updated with joint eye examination guidelines [4, 7].

Regarding congenital glaucoma (CG), physicians’ recognition of the cardinal signs was borderline.

Although a hazy cornea, large cornea, and watering eye (51.4%, 43.9%, and 39.2%, respectively) were recognized to some extent, other unrelated symptoms such as leukocoria and eye redness were mistakenly reported to be CG presentations. While some cases could be missed because of inadequate knowledge, most physicians (n = 145, 98%) were willing to promptly refer a child with CG to an ophthalmologist for further assessment. In addition, all physicians (n = 148, 100%) showed an outstanding awareness of the risk factors for ROP in newborns. In comparison, a Saudi study conducted in the Al-Qassim region demonstrated a lack of knowledge, whereby 50% of the pediatricians had poor knowledge of ROP [9]. Even though early ROP screening is a crucial step in preventing its consequences, only 36.5% of participants knew that it should be done 4–6 weeks after birth or at the postmenstrual age of 32 weeks, whichever comes later. This percentage was comparable to that reported in the Saudi study conducted in Tabuk by Albalawi et al.; however, it was considered very low compared to that reported in the Jordanian study wherein ~ 75% of participants knew the correct timing of ROP screening [8, 10].

Eye examinations should be performed right from the neonatal period and during each follow-up visit. According to the American Academy of Ophthalmology, at the age of 12–36 months, children should have at least one eye screening [4]. When participants were asked about when an ophthalmologist should see a child, 68% responded that a child should undergo vision screening at least once before kindergarten, 24% said that there is no need for one if there are no symptoms of an eye disorder, and only 2% of them did not know when a child should be screened. All newborns and children during their regular well-baby visits should be routinely screened; however, 56 of our participants believed (inaccurately) that this should be done only by an ophthalmologist. This result was similar to that of a Jordanian study in which the authors highly recommended that hospitals should invest in training their staff and instill the importance of early screening and proper timing in referring children to ophthalmologists for further management [8].

Regarding the practices of our pediatricians and family medicine physicians, ~ 81% of our participants performed ophthalmic examinations for children. Some doctors fail to do it because they did not have the required knowledge, competence, time, or equipment. Others could not because the children in question were uncooperative, while others did not do so because thought it was not related to their specialty. Although the assessment of the red reflex is considered a major part of the newborn’s first examination and well-baby visits, 24.3% of our participants failed to do it, which is a lower percentage than that reported in a study conducted in the US wherein only 5% reported that they do not check for the red reflex [14].

This study demonstrated that most (64.8%) junior residents and staff physicians had unsatisfactory.

knowledge (Table 5). This knowledge deficiency might be attributed to inadequate teaching and training during residency. Thus, it is necessary to evaluate the pediatrics and family medicine residency curriculum to ensure that such topics are adequately covered. On the contrary, consultants, associate consultants, specialists, and fellows had satisfactory knowledge, at least to some extent. Differences in the amount of work experience and exposure may explain such differences between senior and junior physicians.

To our knowledge, this is the first study conducted in Saudi Arabia to assess the knowledge and attitude of pediatricians and family physicians toward multiple pediatric eye diseases. Our findings may provide helpful insight into how to plan future teaching. The major study limitation is that the survey was self-reported; hence, there might have been some recall bias.

## Conclusion

In this study, knowledge of eye diseases among participating doctors was reported to be inadequate, especially among residents and staff physicians of both specialties (pediatrics and family medicine). Moreover, consultants, associate consultants, specialists, and fellows demonstrated satisfactory knowledge and skills. Therefore, more intensive ophthalmological educational activities and training, coupled with in-depth assessments during residency, are recommended. Additionally, the residency curriculum needs to be re-evaluated to include these topics right from the beginning of the various programs. Residents should be under supervision when dealing with pediatric cases with ophthalmological complaints since they demonstrated unsatisfactory knowledge. Overall, awareness should be raised, and all family physicians and pediatricians are encouraged to perform thorough eye examinations, given that they are first-contact physicians.

## Electronic supplementary material

Below is the link to the electronic supplementary material.

Additional File 1

## Data Availability

All data generated or analyzed in this study are included in this article. Further inquiries can be directed to the corresponding author.

## List of abbreviations

CG: congenital glaucoma
MNGHA-WR: Ministry of National Guard Health Affairs-Western Region
ROP: retinopathy of prematurity
