# Supplementary material for: Knowledge and attitude toward eye disorders in children among pediatricians and family physicians: a survey study
Source: BMC Ophthalmol. 2023 Mar 7;23:90. doi: 10.1186/s12886-023-02832-5 (PMC9990188; doi:10.1186/s12886-023-02832-5)
Supplement: Supplementary file 1 — Additional File 1 [file 12886_2023_2832_MOESM1_ESM.docx]

| Dear colleagues,  We are conducting a study to assess knowledge of and attitude towards eye disorders in children among paediatricians and family physicians in the Ministry of National Guard Health Affairs, Western Region, Saudi Arabia.  We kindly ask you to fill this brief questionnaire. The information gathered will be confidential and used only for academic purposes, and to improve health services in our country.  Please feel free to participate in this study or to decline. If you are willing to participate, you are kindly requested to append your signature below, Thank you for your cooperation.  Dr. Hashim Almarzogi - Assistant Professor, paediatric ophthalmologist.  Dr. Amer M. Alghamdi - Family Medicine Resident  Dr. Wejdan Alnahdi - Medical Intern  Ms. Reem M. Hersi – 6th Year Medical Student  Ms. Nada K. Naaman– 6th Year Medical Student  Declaration  I have read and understood the above explanation, and I am willing to participate in the study voluntarily.  Signature:  Date: / /2021 |
| --- |

| **Part I:** |
| --- |
| 1. Age: _______ 2. Gender: 3. Male 4. Female 5. You are currently practicing as 6. Pediatric Consultant/Assistant Consultant 7. Pediatric Fellow/specialist 8. Pediatric Staff Physician 9. Pediatric resident 10. Family Medicine Consultant/Assistant Consultant 11. Family Medicine Fellow/specialist 12. Pediatric Staff Physician 13. Family medicine resident 14. At which year of residency training you are in (If applicable)? R_________ 15. How long have you been practicing as a pediatrician/family physician? _________ 16. Have you ever participated in a workshop/conference concerning various eye conditions in children? 17. Yes 18. No |
| **Part II:** *(** You can select more than one option)* |
| 1. **** When should an ophthalmologist see a child?** 2. No need if there are no symptoms of an eye disorder. 3. All newborns. 4. During regular well baby visits 5. Should have vision screening at least once before going to kindergarten 6. I don’t know 7. **** Which of the following can cause red painful eye disease in children?** 8. Conjunctivitis 9. Allergy 10. Uveitis 11. Corneal abrasion/trauma 12. Cataract 13. Glaucoma 14. Squint 15. **** Which of the following can cause leukocoria (white pupil reflex)?** 16. Cataract 17. Glaucoma 18. Retinoblastoma 19. Advance retinal disorder 20. **Leukocoria (white pupil reflex) could be?** 21. Sight threatening 22. Life threatening 23. Normal variation between children 24. **Children of any age may have refractive errors and may need glasses.** 25. True 26. False 27. I don’t know 28. **Refractive errors can cause squint.** 29. True 30. False 31. I don’t know 32. **** Which of the following gives a clue that a child may have TRUE squint?** 33. Eye deviation 34. Face turn 35. Anomalous head posture 36. Epicanthal folds 37. Wide nasal bridge 38. **** What are the concerns about a child with squint?** 39. Cosmetically not acceptable 40. Amblyopia 41. Underlying central cause 42. **Squint can be treated by?** 43. Glasses 44. Surgical repair 45. Spontaneously resolving as a child grows 46. **** Which of the following is a sign of congenital glaucoma?** 47. Watering 48. Leukocoria 49. Large cornea 50. Hazy cornea 51. Red eye 52. **Which of the following may be a Risk factors of ROP (Retinopathy of prematurity)?** 53. Birth weight < 1500 grams 54. GA less than or equal to 32 weeks 55. Premature baby with comorbidities 56. All of the above 57. **Do you perform eye examination in children?** 58. Yes 59. No (If no please move to question 15) 60. **How often do you do eye examination?** 61. When caregiver reports their child has an eye complaint. 62. At birth 63. Routinely with every child’s examination 64. Once a year 65. Other:______________ 66. **** What test do you do?** 67. Red reflex 68. Visual acuity 69. Fundoscopic examination 70. Extraocular muscles motility 71. **** If no, why?** 72. Don’t have enough time 73. No equipment 74. Don’t know how to 75. Children are uncooperative 76. It is not relevant to my profession 77. **How do you manage a child with painful red eye?** 78. Refer immediately to ophthalmologist 79. Give eye drops and refer immediately 80. Give eye drops and if no improvement after 3 days refer to ophthalmologist 81. Others, indicate please ________________   * If you chose "eye drop" please specify the type:__________   1. **How do you manage a child with leukocoria?** 2. Refer to ophthalmologist immediately 3. Give eye drops, specify the eye drop ______ 4. Follow up and if no improvement refers to ophthalmologist 5. Others, indicate please _________________ 6. **How do you manage a child with neonatal conjunctivitis (ophthalmia neonatorum)?**    1. Refer to an ophthalmologist immediately    2. Give eye drops, specify the eye drop____________    3. Follow up and if no improvement, refer to an ophthalmologist    4. Others, indicate please________________________ 7. **How do you manage a child with squint?** 8. Refer to ophthalmologist immediately 9. Give eye drops, specify the eye drop ____________ 10. Follow up and if no improvement refers to ophthalmologist 11. Brain imaging 12. Others, indicate please ___________________________________ 13. **How do you manage a child with congenital glaucoma?** 14. Refer to ophthalmologist immediately 15. Give eye drops, specify the eye drop ___________________ 16. Follow up and if no improvement refers to ophthalmologist 17. Others, indicate please ____________________ 18. **When should a child with congenital cataract be referred to an eye care professional?**     1. When vision drops     2. When caregiver request referral     3. Immediately     4. Others, indicate please______________________ 19. **When will you refer a premature baby for Retinopathy of Prematurity screening?** 20. When discharge from NICU 21. At 4-6 weeks after birth or at 32 weeks, whichever later 22. At birth 23. I do not know |
| 1. **Your training adequately equips you to diagnose, manage, and refer children with eye diseases.**    1. Agree    2. Disagree |
